# Autophagy in Cancer: Context-Dependent Regulation and Precision Nanomedicine-Enabled Therapeutic Targeting

**DOI:** 10.3390/biomedicines14020416

**Published:** 2026-02-12

**Authors:** Yuzhi Lu, Ang Li, Andong Liu, Meng Li, Meng Wang

**Affiliations:** 1 Department of Respiratory and Critical Care Medicine, The Central Hospital of Wuhan, Tongji Medical College, Huazhong University of Science and Technology, Wuhan 430030, China; awhlyz@163.com; 2 Department of Human Anatomy, School of Basic Medicine, Tongji Medical College, Huazhong University of Science and Technology, Wuhan 430030, China; u202313940@hust.edu.cn (A.L.); liuandong@hust.edu.cn (A.L.); 3 Institute of Hematology, Union Hospital, Tongji Medical College, Huazhong University of Science and Technology, Wuhan 430022, China

**Keywords:** autophagy, context-dependent regulation, TME, precision nanomedicine, cancer therapy

## Abstract

Autophagy is a highly conserved cellular degradation process essential for maintaining cellular homeostasis, yet its role in cancer is fundamentally context dependent. Increasing evidence indicates that autophagy suppresses tumor initiation by preserving genomic and metabolic integrity, while paradoxically supporting tumor progression, therapy resistance, and immune evasion at advanced stages. This functional duality presents a major challenge for therapeutic targeting and largely reflects the spatiotemporal heterogeneity of autophagy regulation across tumor stages, cancer cell subpopulations, and the tumor microenvironment (TME). In this review, we argue that autophagy-related proteins should be conceptualized as context-dependent therapeutic nodes rather than universally actionable targets. We systematically examine key autophagy regulators, including Beclin-1, p62/SQSTM1, mTOR, and p53, and analyze how their functions are shaped by tumor stage, genetic background, and microenvironmental cues such as hypoxia, immune pressure, and stromal interactions. We further highlight the pivotal role of the TME in determining autophagy dependency and therapeutic vulnerability, providing mechanistic insight into why autophagy modulation without microenvironmental consideration often yields inconsistent outcomes. From a precision medicine perspective, we discuss how nanotechnology-based delivery systems enable spatially and temporally controlled modulation of autophagy, thereby addressing intratumoral heterogeneity and reducing systemic toxicity. By integrating molecular profiling, TME characteristics, and nanomedicine-enabled targeting strategies, this review outlines a rational framework for exploiting autophagy in cancer therapy. Together, these insights provide a foundation for the development of context-aware, autophagy-targeted interventions and advance the pursuit of more effective and personalized cancer treatments.

## 1. Introduction

**Figure 1:**
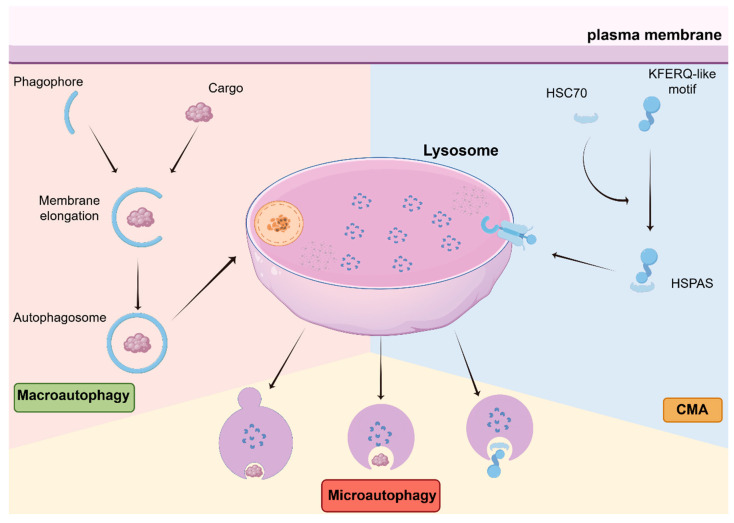
Three forms of autophagy: macroautophagy, microautophagy and CMA. In macroautophagy, the phagophore elongates to form the autophagosome, which then fuses with the lysosome for cargo degradation. Microautophagy involves the direct engulfment of cellular material by the lysosome. CMA is characterized by the selective recognition of substrates with KFERQ-like motifs by the chaperone protein HSC70, which then mediates their translocation into the lysosome for degradation.

Autophagy is an evolutionarily conserved intracellular degradation system that plays a fundamental role in maintaining cellular homeostasis. Since its initial characterization more than half a century ago, autophagy has been recognized as a highly coordinated and multi-layered process encompassing distinct forms, including macroautophagy, microautophagy, and chaperone-mediated autophagy (CMA) (Figure 1), each governed by specialized molecular machinery and regulatory networks [1,2]. This molecular complexity underlies the broad physiological relevance of autophagy in development, metabolism, and disease.

In cancer, autophagy has long been viewed as a paradoxical process. Early genetic and functional studies established a tumor-suppressive role for basal autophagy through the maintenance of genomic stability, proteostasis, and cellular quality control [3,4]. In contrast, subsequent work demonstrated that established tumors frequently exploit autophagy as an adaptive mechanism to survive metabolic stress, hypoxia, and therapeutic insults [5,6]. As a result, both autophagy activation and inhibition have been reported to produce beneficial or detrimental effects depending on experimental context, leading to inconsistent outcomes in preclinical models and clinical trials [7,8,9].

A central explanation for these discrepancies lies in the stage- and context-dependent nature of autophagy in cancer. Autophagic activity is dynamically regulated throughout tumor evolution and varies substantially among genetically and metabolically heterogeneous cancer cell populations [10]. Moreover, autophagy is not confined to malignant cells. Immune cells, endothelial cells, and cancer-associated fibroblasts (CAFs) within the TME exhibit distinct autophagy programs that critically influence tumor growth, immune surveillance, and therapeutic resistance [11,12]. Consequently, autophagy-targeted interventions inevitably affect multiple cellular compartments, with therapeutic outcomes determined by the integrated response of the tumor ecosystem rather than cancer cells alone.

The TME imposes additional selective pressures that actively rewire autophagy pathways. Hypoxia and nutrient deprivation robustly induce autophagy in both tumor and immune cells, reshaping immune responses and treatment sensitivity [11,12]. Notably, recent studies have demonstrated that autophagy-mediated degradation of major histocompatibility complex class I (MHC-I) molecules facilitates tumor immune evasion, whereas autophagy inhibition can restore antigen presentation and enhance immunotherapy efficacy [13]. These findings underscore the critical importance of microenvironmental context in determining whether autophagy restrains or promotes tumor progression.

Precision medicine provides a conceptual framework for addressing the context dependency of autophagy by integrating molecular profiling, tumor staging, and microenvironmental features to guide therapeutic decision-making [14,15]. Within this framework, nanotechnology-based drug delivery systems offer unique advantages for spatially and temporally controlled modulation of autophagy. Nanocarriers can enhance tumor-specific drug accumulation, enable cell-type-selective targeting, and fine-tune autophagic responses while minimizing systemic toxicity [16]. Emerging nanomedicine strategies have demonstrated the feasibility of modulating autophagy-related pathways, including Beclin-1, mTOR, and p53 signaling in a context-aware manner [17,18,19].

In this review, we propose that autophagy-related proteins should be redefined as context-dependent therapeutic targets rather than universal intervention points. By synthesizing current evidence on the stage- and microenvironment-specific roles of key autophagy regulators and discussing how precision nanomedicine enables spatiotemporal control of autophagy within heterogeneous tumors, we aim to provide a rational framework for exploiting autophagy in personalized cancer therapy.

## 2. Autophagy as a Stage- and Context-Dependent Process in Cancer

Autophagy functions as a dynamic and adaptive process throughout tumor development, exerting distinct and sometimes opposing roles at different stages of cancer progression. During early tumorigenesis, basal autophagy contributes to tumor suppression by maintaining cellular homeostasis, limiting oxidative stress, and preventing the accumulation of damaged organelles and oncogenic protein aggregates. Genetic or functional disruption of core autophagy machinery has been shown to promote genomic instability and malignant transformation, supporting the protective role of autophagy during the initial phases of tumor development.

As tumors progress, however, cancer cells are increasingly exposed to hostile microenvironmental conditions characterized by hypoxia, nutrient deprivation, and therapeutic stress. Under these circumstances, autophagy is frequently co-opted as an adaptive survival mechanism that supports metabolic flexibility and stress tolerance. This transition gives rise to a state often described as “autophagy addiction,” in which advanced tumors rely on sustained autophagic flux to cope with bioenergetic and proteotoxic stress. In this setting, autophagy inhibition can sensitize tumors to chemotherapy, radiotherapy, or targeted therapies, although therapeutic efficacy remains highly context dependent [7,20,21].

Intratumoral heterogeneity further complicates efforts to therapeutically target autophagy. Cancer cell subpopulations within the same tumor differ substantially in genetic alterations, metabolic requirements, and stress adaptability, resulting in variable dependence on autophagy. Consequently, global modulation of autophagy may eliminate vulnerable cell populations while inadvertently selecting for more aggressive and therapy-resistant clones. Moreover, autophagy can either promote survival or trigger non-apoptotic cell death depending on the genetic and signaling context, underscoring the limitations of uniform therapeutic strategies [22].

Importantly, autophagy is not restricted to malignant cells but operates across multiple cellular compartments within the TME. In immune cells, autophagy regulates antigen presentation, cytokine secretion, and cell viability, thereby shaping anti-tumor immunity. In stromal cells, particularly CAFs, autophagy supports metabolic coupling with cancer cells and facilitates tumor recovery following therapeutic stress, including radiotherapy [23,24]. These non-cell-autonomous effects highlight that the net impact of autophagy modulation reflects the integrated response of the tumor ecosystem rather than cancer cells alone.

Taken together, these observations emphasize that autophagy in cancer should be viewed as a stage-dependent and context-specific process rather than a static pathway. Effective therapeutic exploitation of autophagy therefore requires precise consideration of tumor stage, cellular heterogeneity, and microenvironmental conditions, providing a strong rationale for integrating autophagy-targeted interventions with precision medicine strategies.

## 3. Context-Dependent Roles of Key Autophagy Regulators in Cancer

**Figure 2:**
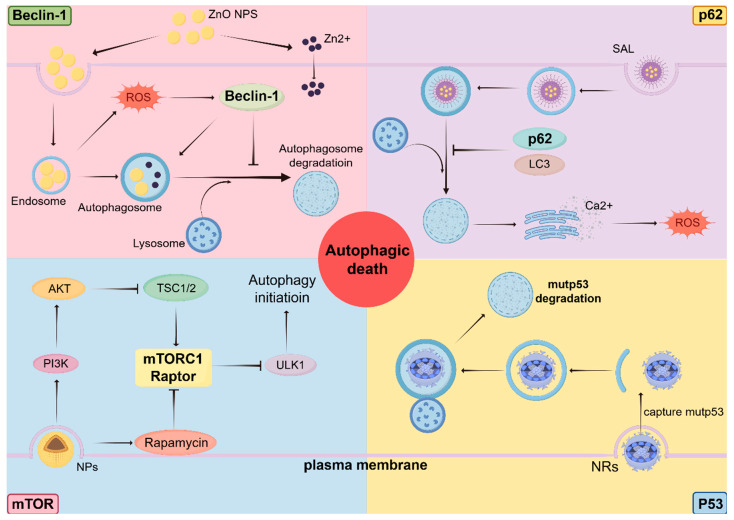
Context-dependent roles of key autophagy regulators in cancer. Upper left: Beclin-1-dependent autophagy modulation by nanoparticles. Upper right: p62-mediated selective autophagy and stress signaling. Bottom left: mTOR signaling and autophagy initiation. Bottom right: Autophagy-mediated degradation of mutant p53.

Autophagy-related proteins are often discussed as discrete molecular targets; however, accumulating evidence indicates that their biological functions and therapeutic relevance are profoundly shaped by tumor stage, genetic background, and microenvironmental context. Central regulators such as Beclin-1, p62/SQSTM1, mTOR, and p53 occupy nodal positions at the intersection of autophagy, metabolism, stress responses, and cell fate determination. Rather than functioning as linear components of a uniform pathway, these proteins act as context-sensitive signaling hubs whose modulation can yield divergent therapeutic outcomes (Figure 2).

### 3.1. Beclin-1: Tumor Suppressor, Stress Adaptor, or Therapeutic Switch

Beclin-1, encoded by *BECN1*, was among the first autophagy-related proteins implicated in cancer and is frequently described as a haploinsufficient tumor suppressor [25]. Early studies demonstrated that reduced *BECN1* expression promotes tumor initiation, supporting the concept that basal autophagy restrains malignant transformation by preserving cellular homeostasis [26]. These findings positioned Beclin-1 as a canonical tumor-suppressive regulator during early-stage tumorigenesis.

Subsequent work, however, revealed a more nuanced and context-dependent role for Beclin-1 in established tumors. Beclin-1 participates in multiple protein complexes that integrate signals from nutrient availability, growth factor signaling, and cellular stress, thereby fine-tuning autophagic flux [27]. In metabolically stressed or hypoxic tumor environments, Beclin-1-dependent autophagy enables cancer cells to maintain bioenergetic balance and limit proteotoxic damage, effectively functioning as a stress-adaptive mechanism that supports tumor progression and therapy resistance [3,10].

Recent nanomedicine-based studies further illustrate the therapeutic ambiguity of targeting Beclin-1. Nanoparticle-mediated modulation of Beclin-1 signaling has been reported to induce autophagic cell death or suppress tumor growth in a cancer type- and context-dependent manner [28,29,30]. Conversely, targeted suppression of *BECN1* using a peptide-conjugated poly (β-amino ester) can inhibit autophagy and restrain tumor progression in autophagy-dependent models [31]. Collectively, these findings underscore that Beclin-1 cannot be uniformly classified as either a beneficial or detrimental therapeutic target; instead, its relevance depends critically on the timing, location, and mode of autophagy modulation.

### 3.2. p62/SQSTM1: Autophagy Flux Sensor and Oncogenic Signaling Hub

p62/SQSTM1 occupies a unique position at the interface between selective autophagy and oncogenic signaling. As an autophagy receptor, p62 mediates the delivery of ubiquitinated cargo to autophagosomes, while its own degradation serves as a functional indicator of autophagic flux [32]. Accumulation of p62 is commonly observed in autophagy-deficient settings and has been linked to tumor-promoting signaling through activation of NRF2, NF-κB, and mTOR pathways [33].

Paradoxically, p62 also cooperates with intact autophagy to promote tumor progression. Rather than acting solely as a passive substrate, p62 functions as a signaling scaffold that integrates stress responses, metabolic reprogramming, and survival pathways. In tumors with functional autophagic flux, dynamic turnover of p62 supports adaptation to oxidative and metabolic stress, whereas disruption of this balance can shift p62 toward a driver of oncogenic signaling.

These dual roles complicate therapeutic strategies targeting p62. Autophagy inhibition may lead to pathological accumulation of p62, inadvertently enhancing tumor-promoting pathways. Conversely, restoring autophagic degradation of p62 can suppress oncogenic signaling in selected contexts [34]. Thus, p62 exemplifies a key principle in autophagy-targeted therapy: therapeutic outcome is determined by modulation of autophagic flux rather than static alteration of individual pathway components.

### 3.3. mTOR: Metabolic Gatekeeper Linking Autophagy and Therapeutic Vulnerability

mTOR serves as a master regulator of cellular metabolism and a central inhibitory node controlling autophagy initiation [35]. Hyperactivation of the PI3K/AKT/mTOR axis is a hallmark of many cancers and contributes to uncontrolled proliferation, metabolic rewiring, and therapeutic resistance [36]. Accordingly, pharmacological inhibition of mTOR has been widely explored as a strategy to induce autophagy and suppress tumor growth.

Despite its conceptual appeal, mTOR inhibition produces heterogeneous outcomes across tumor types and treatment contexts. In some settings, mTOR inhibition-induced autophagy promotes cancer cell survival by alleviating metabolic stress and limiting therapy-induced damage. In others, excessive or dysregulated autophagy can trigger non-apoptotic cell death or sensitize tumors to chemotherapy and radiotherapy [37]. These divergent responses reflect differences in tumor stage, metabolic dependency, and co-occurring genetic alterations.

Nanoparticle-based delivery systems provide new opportunities to refine mTOR-targeted autophagy modulation. Nanocarriers encapsulating mTOR inhibitors or autophagy modulators enable controlled drug release, enhanced tumor accumulation, and reduced systemic toxicity [38,39]. Importantly, precision delivery allows selective exploitation of autophagy dependency in advanced tumors while limiting protective autophagy in normal tissues, reinforcing the concept that mTOR represents a context-sensitive therapeutic node rather than a universal autophagy switch.

### 3.4. p53: Genotype-Dependent Regulator of Autophagy and Therapy Response

p53 plays a multifaceted role in autophagy regulation, acting as either an inducer or suppressor depending on its mutation status, subcellular localization, and signaling context [40]. Wild-type p53 can promote autophagy through transcriptional activation of autophagy-related genes and metabolic stress pathways, contributing to tumor suppression during early disease stages. In contrast, cytoplasmic p53 has been reported to inhibit autophagy, further highlighting the complexity of p53-mediated regulation.

In p53-deficient or mutant tumors, autophagy regulation is frequently rewired, altering therapeutic vulnerabilities. Mutant p53 can cooperate with autophagy to promote tumor survival and chemoresistance, whereas restoration of wild-type p53 activity may sensitize tumors to autophagy modulation [41]. Recent nanomedicine-based approaches have leveraged this dependency by delivering wild-type p53 or promoting selective degradation of mutant p53 via autophagy-related mechanisms, thereby enhancing therapeutic efficacy [42,43].

These findings illustrate that p53 status is a critical determinant of whether autophagy activation or inhibition is therapeutically advantageous. As such, p53 exemplifies the importance of genotype-informed strategies when integrating autophagy modulation with precision delivery platforms.

### 3.5. Implications for Precision Autophagy Targeting

Collectively, these examples demonstrate that key autophagy regulators function as context-dependent signaling hubs rather than linear drug targets. The therapeutic impact of modulating Beclin-1, p62, mTOR, or p53 is dictated by tumor stage, genetic background, metabolic state, and microenvironmental pressures. Failure to account for these variables helps explain the inconsistent outcomes observed in clinical attempts to target autophagy.

Precision medicine approaches—particularly when combined with nanotechnology-enabled delivery systems—offer a viable path forward by enabling spatially and temporally controlled modulation of autophagy in defined cellular contexts. A clear understanding of the context-dependent biology of autophagy regulators is therefore essential for transforming autophagy from a paradoxical pathway into a rational and actionable therapeutic opportunity.

## 4. TME as a Determinant of Autophagy Dependency

Autophagy in cancer cannot be fully understood without considering the TME, which imposes dynamic and heterogeneous stresses that profoundly shape autophagy dependency and therapeutic vulnerability. The TME comprises a complex and evolving network of malignant cells, immune infiltrates, stromal components, vasculature, and extracellular matrix, all of which experience fluctuating oxygen and nutrient availability as well as immune and therapeutic pressures [44,45,46]. These microenvironmental conditions act as powerful regulators of autophagy, rendering its functional consequences highly context-specific.

### 4.1. Hypoxia and Metabolic Stress as Drivers of Autophagy Rewiring

Hypoxia and nutrient deprivation are defining features of solid tumors and among the most potent inducers of autophagy. Oxygen limitation activates autophagy through multiple mechanisms, including suppression of mTOR signaling and engagement of stress-responsive pathways, enabling tumor cells to maintain energy homeostasis under adverse conditions [47]. Autophagy-mediated recycling of intracellular components supports metabolic flexibility and promotes survival in poorly vascularized tumor regions.

Beyond cancer cells, hypoxia-driven autophagy also exerts important effects on immune cell function within the TME. In immune populations exposed to low oxygen tension, autophagy modulates cytokine production, antigen processing, and cell survival, thereby reshaping anti-tumor immune responses [11]. These observations highlight the dual role of hypoxia-induced autophagy: while facilitating tumor cell adaptation, it simultaneously influences immune surveillance in a context-dependent manner.

### 4.2. Autophagy-Mediated Immune Evasion and Immunotherapy Response

Recent studies have uncovered a direct mechanistic link between autophagy and tumor immune evasion. Autophagy can selectively degrade components of the antigen presentation machinery, most notably MHC-I molecules, thereby reducing tumor recognition by cytotoxic T lymphocytes [13]. This mechanism has been particularly well characterized in pancreatic cancer, where autophagy-mediated MHC-I degradation contributes to immune escape and resistance to immunotherapy.

Conversely, inhibition of autophagy has been shown to restore surface expression of MHC-I molecules, enhance antigen presentation, and improve the efficacy of immune checkpoint blockade in selected tumor models [48]. These findings underscore that autophagy modulation can reprogram tumor immune interactions, but only within specific immunological and microenvironmental contexts. Autophagy should therefore be viewed not merely as a cell-autonomous survival pathway but also as a regulator of tumor immunogenicity.

### 4.3. Autophagy in Cancer-Associated Fibroblasts and Stromal Support

CAFs constitute a major stromal component of the TME and play a central role in tumor progression and therapy resistance. Emerging evidence indicates that CAFs exhibit elevated autophagic activity, which supports metabolic coupling between stromal and cancer cells [49]. Through autophagy-dependent recycling processes, CAFs supply metabolites and growth-supportive factors that fuel tumor growth and facilitate recovery following therapeutic stress.

Autophagy in CAFs has been particularly implicated in resistance to radiotherapy and chemotherapy. Experimental models demonstrate that autophagy-competent CAFs promote the survival and regrowth of irradiated cancer cells, whereas disruption of stromal autophagy sensitizes tumors to treatment. These findings reveal a non-cell-autonomous dimension of autophagy dependency and suggest that targeting autophagy exclusively in malignant cells may be insufficient to overcome therapy resistance.

### 4.4. Therapeutic Stress, Adaptive Autophagy, and Resistance Mechanisms

Therapeutic interventions themselves represent a major selective pressure within the TME and frequently induce adaptive autophagy as a cytoprotective response. Chemotherapy, radiotherapy, and targeted therapies can activate autophagy through mechanisms involving DNA damage, oxidative stress, and metabolic disruption [50,51]. While such stress-induced autophagy may initially limit tumor growth, it often contributes to the emergence of treatment resistance by enabling cancer cells to withstand cytotoxic insults.

Importantly, the contribution of autophagy to therapy resistance is strongly influenced by microenvironmental factors. Hypoxic tumor regions, for example, often display heightened autophagy dependency following treatment, whereas well-oxygenated areas may respond differently. This spatial heterogeneity further complicates uniform therapeutic strategies and underscores the need for context-aware modulation of autophagy.

### 4.5. Targeting TME-Driven Autophagy: Implications for Precision Therapy

Collectively, these findings establish the TME as a decisive determinant of autophagy dependency in cancer. Hypoxia, immune interactions, stromal support, and therapy-induced stress converge to reprogram autophagy across multiple cellular compartments, dictating whether autophagy restrains tumor growth or promotes tumor persistence. Therapeutic strategies that fail to account for this microenvironmental complexity are therefore unlikely to achieve durable clinical benefit.

Precision medicine approaches that integrate tumor stage, immune profiling, and microenvironmental characteristics provide a rational framework for exploiting TME-driven autophagy vulnerabilities [52]. In this context, nanotechnology-based delivery systems offer a powerful means to selectively modulate autophagy within specific regions or cell types of the TME, thereby enhancing therapeutic efficacy while minimizing off-target effects [53]. Understanding and targeting TME-driven autophagy thus represents a critical step toward the rational design of personalized, autophagy-informed cancer therapies.

### 4.6. Therapeutic Agents Targeting Autophagy

A growing number of pharmacological agents have been developed to modulate autophagy at different stages of the autophagic process, reflecting the increasing recognition of autophagy as a therapeutically actionable pathway in cancer. These agents broadly target autophagy initiation, autophagosome formation, lysosomal fusion, or autophagic flux completion, and have been evaluated across preclinical models and clinical trials.

Among the most extensively investigated autophagy inhibitors are lysosomal inhibitors, including chloroquine (CQ) and hydroxychloroquine (HCQ), which impair autophagosome–lysosome fusion by increasing lysosomal pH [54]. These agents have advanced furthest clinically and have been tested in multiple phase I/II clinical trials in combination with chemotherapy, radiotherapy, or targeted agents in solid tumors and hematological malignancies [55,56]. In addition to lysosomal inhibitors, upstream autophagy regulators have emerged as alternative therapeutic targets. Inhibitors of ULK1/2, VPS34, and Beclin-1 complexes have demonstrated potent autophagy suppression in preclinical cancer models [57,58]. Conversely, autophagy inducers, including mTOR inhibitors such as rapamycin and its analogs, have exhibited context-dependent anti-tumor or pro-survival effects [59,60]. While autophagy induction can promote autophagic cell death in selected tumor settings, in many cancers it enhances metabolic adaptation and therapeutic resistance, complicating its clinical exploitation (Table 1).

Collectively, although autophagy-targeting agents have demonstrated compelling biological activity, their clinical translation remains constrained by limited tumor selectivity, narrow therapeutic windows, and pronounced context dependency. These challenges provide a strong rationale for the development of advanced drug delivery strategies particularly nanocarrier-based systems to enhance tumor localization, reduce systemic exposure, and enable rational combination therapies tailored to tumor microenvironmental features [66].

## 5. Precision Nanomedicine for Spatiotemporal Modulation of Autophagy

The context-dependent nature of autophagy presents a fundamental obstacle to therapeutic intervention, as indiscriminate activation or inhibition of autophagy often produces unpredictable and even opposing effects. Precision nanomedicine offers a compelling strategy to address this challenge by enabling spatially and temporally controlled modulation of autophagy, improving tumor specificity, and minimizing systemic toxicity [67,68,69]. By integrating targeted delivery, controlled release, and microenvironment-responsive design, nanotechnology provides a practical framework for translating autophagy biology into personalized cancer therapy (Table 2).

### 5.1. Nanomedicine and Nanoscale Drug Delivery Paradigms

Nanomedicine encompasses the application of nanoscale materials for disease diagnosis and therapy, with cancer drug delivery representing one of its most mature and clinically explored domains [82]. Numerous studies have demonstrated that nanoscale formulations can significantly alter the pharmacokinetics and biodistribution of anticancer agents, leading to improved therapeutic indices compared with conventional small-molecule drugs [83,84,85].

A broad spectrum of nanocarriers has been investigated for oncological applications, including liposomes, polymeric nanoparticles, polymeric micelles, SLNs, albumin-based nanoparticles, inorganic nanoparticles and hybrid nanostructures [86]. Clinically approved examples, such as liposomal doxorubicin and albumin-bound paclitaxel, have provided that nanoscale delivery can reduce systemic toxicity while maintaining or enhancing antitumor efficacy [87]. Preclinical studies further suggest that polymeric and inorganic nanocarriers can be engineered to improve drug stability and intracellular accumulation, particularly for hydrophobic or rapidly metabolized compounds [88].

Nanoscale drug delivery systems can be broadly categorized according to their targeting mechanisms. Passive targeting exploits the EPR effect, a phenomenon arising from the abnormal vasculature and impaired lymphatic drainage characteristic of many solid tumors [89]. Nanoparticles circulating in the bloodstream may preferentially accumulate within tumor tissue due to leaky endothelial junctions, thereby increasing local drug concentrations. While the EPR effect has been widely demonstrated in preclinical models, its magnitude and reliability vary substantially between tumor types, disease stages, and patients, limiting its universal applicability and underscoring the need for additional targeting strategies [90].

To address these limitations, active targeting strategies have been developed in which nanocarriers are functionalized with ligands capable of recognizing tumor-associated receptors. Antibody- or peptide-decorated nanoparticles targeting HER2, EGFR, folate receptor, transferrin receptor, and integrins have been shown to enhance cellular uptake and intracellular drug accumulation in vitro and in vivo [91,92,93,94,95]. Nevertheless, experimental studies also demonstrate that active targeting does not completely override nonspecific biodistribution and remains influenced by receptor heterogeneity, ligand density, and protein corona formation.

Beyond targeting, increasing emphasis has been placed on stimuli-responsive (“smart”) nanomedicine systems, which aim to achieve spatially and temporally controlled drug release [96]. pH-sensitive nanocarriers have been extensively studied to exploit the acidic TME or endolysosomal compartments [97]. Redox-responsive and reactive oxygen species-sensitive formulations further enable intracellular drug activation [98]. In parallel, externally triggered systems such as light, ultrasound, or magnetic-field-responsive nanoparticles have demonstrated the feasibility of site-specific drug release following localized stimulation [99,100,101]. Collectively, these studies provide experimental evidence that smart nanocarriers can improve therapeutic specificity and reduce systemic exposure.

Importantly, there are also many limitations and challenges of nanoscale drug delivery. Variability in tumor vascularization, stromal density, and immune cell infiltration can profoundly affect nanoparticle penetration and retention [102]. In addition, manufacturing complexity, scalability, long-term safety, and regulatory considerations remain significant barriers to clinical translation [103]. These factors underscore that nanomedicine does not represent a universally superior solution, but rather a set of tools whose effectiveness depends on biological and clinical context. Within this evidence-based framework, precision nanomedicine can be viewed as the rational integration of nanoscale formulation strategies with tumor-specific characteristics, including molecular profile, microenvironmental features, and disease stage. Rather than assuming uniform benefit, precision nanomedicine emphasizes selecting or designing nanocarriers that align with defined therapeutic objectives. This concept provides a logical transition to subsequent sections, where nanotechnology-based strategies are discussed in relation to the context-dependent modulation of autophagy in cancer.

### 5.2. Targeted Nanocarriers for Modulating Beclin-1–Dependent Autophagy

A growing body of evidence demonstrates that nanomedicine-based approaches can effectively modulate Beclin-1-dependent autophagy in cancer [104]. Liposomal and polymeric nanoparticles loaded with autophagy-modulating agents have been shown to induce Beclin-1-associated autophagic responses, leading to tumor growth inhibition or autophagic cell death in selected tumor models [105]. In lung and ovarian cancer systems, nanoparticle formulations that enhance Beclin-1-mediated autophagy have been reported to exert anti-tumor effects through excessive stress induction or non-apoptotic cell death pathways [28,29].

Conversely, nanocarriers delivering microRNAs or small interfering RNAs targeting *BECN1* have been used to suppress autophagy in autophagy-dependent tumors, resulting in reduced tumor progression and enhanced treatment sensitivity [106]. These contrasting strategies reinforce a central concept of this review: Beclin-1 functions as a context-dependent therapeutic switch, and nanotechnology enables its bidirectional modulation in a tumor- and stage-specific manner.

### 5.3. Nanomedicine-Enabled Control of mTOR Signaling and Autophagy Flux

Given its central role in autophagy regulation, mTOR has been extensively explored as a therapeutic target in cancer. Nanoparticle-mediated delivery of mTOR inhibitors has been shown to improve drug stability, pharmacokinetics, and tumor accumulation compared with free agents [107]. Polymeric nanoparticles and albumin-bound formulations encapsulating sirolimus or related compounds exemplify this strategy and have demonstrated enhanced anti-tumor efficacy in preclinical and clinical settings [108].

Beyond improving drug delivery, advanced nanocarriers have been engineered to fine-tune autophagy flux by modulating upstream signaling pathways and lysosomal function [109]. Such systems enable controlled induction or inhibition of autophagy depending on tumor dependency and treatment context. Importantly, nanomedicine-based mTOR modulation can be integrated with chemotherapy, radiotherapy, or immunotherapy to exploit autophagy-mediated vulnerabilities while limiting adaptive resistance.

### 5.4. Nanoparticle-Based Strategies Targeting p53-Autophagy Interplay

The interplay between p53 status and autophagy regulation offers unique opportunities for precision nanomedicine. Nanoparticle-mediated delivery of wild-type p53 has been shown to sensitize p53-deficient tumors to chemotherapy and targeted therapies, in part through modulation of autophagy-related signaling pathways [19]. These approaches illustrate how restoration of tumor suppressor function can be coupled with reprogramming of autophagy dependency.

More recently, innovative nanoreceptor systems have been developed to promote selective degradation of mutant p53 by mimicking autophagy receptors, thereby reactivating autophagy-mediated tumor suppression [42]. Such strategies demonstrate the potential of nanotechnology to manipulate autophagy at the level of specific protein–protein interactions rather than through indiscriminate pathway inhibition.

### 5.5. Responsive and Multifunctional Nanocarriers for TME-Adapted Autophagy Modulation

The TME provides distinct physicochemical cues—including acidic pH, hypoxia, and elevated reactive oxygen species—that can be exploited for responsive nanomedicine design. Multifunctional nanocarriers capable of sensing and responding to these cues enable localized modulation of autophagy within specific tumor regions [110].

Recent studies have shown that ultrasmall nanoparticles and metal-based nanomaterials can induce or disrupt autophagy by interfering with autophagosome formation, maturation, or lysosomal function [111]. In parallel, hybrid nanovesicle systems have been developed to modulate autophagy within the TME to enhance anti-tumor immunity, highlighting the potential of combining autophagy regulation with immunomodulatory strategies [112]. These advances underscore that nanotechnology can function not only as a delivery vehicle but also as an active regulator of autophagy dynamics.

### 5.6. Challenges and Perspectives for Clinical Translation

Despite encouraging preclinical progress, several challenges hinder the clinical translation of nanomedicine-based autophagy modulation. These include variability in nanoparticle biodistribution, incomplete understanding of long-term effects on autophagy in non-malignant tissues, and the lack of robust biomarkers to monitor autophagy flux in patients. Moreover, the complexity of autophagy regulation within the TME necessitates careful patient stratification and treatment customization.

Nonetheless, the convergence of precision medicine, nanotechnology, and autophagy biology offers a compelling opportunity to overcome the longstanding paradox of autophagy targeting in cancer. By enabling spatially and cell-type-selective modulation of autophagy, precision nanomedicine provides a rational pathway toward more effective and personalized cancer therapies.

## 6. Integrating Autophagy Targeting with TME Modulation and Combination Therapy

Given the multifaceted and context-dependent roles of autophagy in cancer, increasing evidence suggests that autophagy modulation is unlikely to achieve durable therapeutic benefit as a standalone intervention. Instead, its clinical relevance emerges most clearly when integrated with TME modulation and established treatment modalities, including chemotherapy, radiotherapy, targeted therapy, and immunotherapy. Such combination strategies aim to exploit autophagy-dependent vulnerabilities while limiting adaptive resistance and preserving anti-tumor immune function.

### 6.1. Autophagy Modulation to Overcome Therapy Resistance

Adaptive autophagy is a well-recognized mechanism by which cancer cells tolerate chemotherapy and targeted therapies. Numerous cytotoxic agents and molecular inhibitors induce autophagy as a stress-response pathway, enabling tumor cells to survive therapy-induced DNA damage, oxidative stress, and metabolic disruption [113]. In tumors exhibiting high basal or inducible autophagy, pharmacological or genetic inhibition of autophagy has been shown to enhance sensitivity to chemotherapeutic agents, histone deacetylase inhibitors, and kinase inhibitors. However, the therapeutic impact of autophagy inhibition is strongly influenced by tumor stage and microenvironmental conditions. In advanced tumors characterized by hypoxia and nutrient limitation, autophagy inhibition may preferentially impair stress-adapted cancer cell populations [114]. In contrast, in early-stage or metabolically less constrained tumors, autophagy suppression may offer limited benefit or even disrupt cellular homeostasis. These observations underscore the importance of appropriate patient stratification and treatment timing when combining autophagy modulation with conventional therapies.

### 6.2. Enhancing Radiotherapy Efficacy Through Stromal and Microenvironmental Autophagy Targeting

Radiotherapy imposes substantial stress on both malignant cells and stromal components of the TME, leading to robust activation of autophagy. Notably, CAFs have emerged as key contributors to tumor regrowth following irradiation through autophagy-dependent mechanisms [115]. Autophagic activity in CAFs supports metabolic recycling and paracrine signaling that facilitate post-radiation survival and recovery of tumor cells.

Experimental disruption of stromal autophagy has been shown to impair tumor repair processes and enhance radiosensitivity [116,117]. These findings indicate that effective integration of autophagy modulation with radiotherapy should consider both cancer cell-intrinsic and stromal autophagy programs.

### 6.3. Autophagy and Immunotherapy: Balancing Immune Activation and Immune Evasion

Autophagy plays a central role in shaping anti-tumor immunity by regulating antigen presentation, immune cell survival, and cytokine secretion within the TME [118,119,120]. Recent studies have demonstrated that autophagy-mediated degradation of antigen presentation machinery, including MHC class I molecules, can promote immune evasion, whereas autophagy inhibition may restore tumor immunogenicity and enhance responsiveness to immune checkpoint blockade [121,122].

At the same time, autophagy supports immune cell viability under metabolically stressful conditions, raising concerns that indiscriminate autophagy inhibition could impair immune function. These dual roles suggest that selective or temporally controlled modulation of autophagy—rather than uniform pathway inhibition—may be required to enhance immunotherapeutic efficacy while preserving immune competence.

### 6.4. Nanomedicine-Enabled Combination Strategies Targeting Autophagy and the TME

Nanotechnology-based delivery systems provide a practical platform for integrating autophagy modulation with combination therapies that act on the TME. Several studies have demonstrated that nanocarriers can co-deliver autophagy modulators together with chemotherapeutic agents, immunomodulators, or metabolic inhibitors, resulting in synergistic anti-tumor effects in preclinical models [123,124].

In addition, multifunctional nanocarriers responsive to microenvironmental cues—such as acidic pH, hypoxia, or redox imbalance—enable localized drug release in tumor regions with heightened autophagy dependency [125,126,127]. Rather than serving as universally applicable solutions, these systems illustrate how delivery strategies can be adapted to spatial and metabolic heterogeneity within tumors.

### 6.5. Clinical Considerations and Future Integration Strategies

Despite encouraging preclinical data, clinical integration of autophagy-targeted combination strategies remains challenging. Major barriers include the lack of validated biomarkers to assess autophagy dependency, uncertainty regarding optimal treatment sequencing, and concerns over cumulative toxicity associated with sustained autophagy modulation [128].

Future clinical strategies should therefore emphasize rational trial design informed by tumor stage, molecular profiling, and microenvironmental characteristics. Incorporation of pharmacodynamic biomarkers and adaptive treatment frameworks may allow autophagy modulation to be applied selectively, maximizing therapeutic benefit while minimizing toxicity and resistance.

## 7. Challenges and Future Perspectives

Despite substantial progress in elucidating the molecular mechanisms and functional roles of autophagy in cancer, the clinical translation of autophagy-targeted therapies remains limited. This gap reflects not a lack of biological relevance, but rather the intrinsic complexity and context dependency of autophagy regulation. Addressing these challenges requires a shift away from uniform pathway modulation toward selective, conditional, and context-aware therapeutic strategies.

### 7.1. Defining and Measuring Autophagy Dependency in Patients

One of the most significant barriers to effective autophagy-targeted therapy is the absence of robust biomarkers capable of defining autophagy dependency in human tumors. Autophagy is a highly dynamic, multistep process, and static measurements of autophagy-related proteins such as LC3 or p62 often fail to capture functional autophagic flux, resulting in imprecise patient stratification and heterogeneous clinical outcomes.

Future efforts should focus on dynamic and integrative biomarker strategies, combining molecular signatures, metabolic states, and microenvironmental features. Advances in multi-omics profiling, functional imaging, and real-time assessment of autophagy activity may enable more accurate evaluation of autophagy dependency in clinical samples [128]. Importantly, such approaches should extend beyond cancer cells to include immune and stromal compartments of the TME.

### 7.2. Managing Spatial and Temporal Heterogeneity of Autophagy

Tumor heterogeneity represents a fundamental obstacle to effective autophagy targeting. Autophagy dependency varies across tumor regions, cellular subpopulations, and disease stages, driven by gradients in oxygen availability, nutrient supply, and therapeutic exposure. Consequently, uniform autophagy modulation often produces incomplete or transient responses, facilitating the emergence of resistant subclones.

One promising solution to this challenge is the use of smart drug delivery systems, which enable spatially and temporally controlled modulation of autophagy. Such systems are designed to respond to tumor-associated cues, including acidic pH, hypoxia, elevated reactive oxygen species, or enzyme activity, thereby restricting drug release to regions with heightened autophagy dependency.

By localizing autophagy modulation to specific tumor niches—such as hypoxic or therapy-resistant regions—smart systems offer a strategy to address intratumoral heterogeneity while minimizing off-target effects in normoxic tissues. However, successful implementation will require improved understanding of intratumoral nanoparticle distribution and precise control of release kinetics.

### 7.3. Balancing Anti-Tumor Efficacy with Immune Preservation

Autophagy plays indispensable roles in immune cell survival, antigen presentation, and cytokine regulation, raising important concerns regarding the immunological consequences of autophagy modulation. While autophagy inhibition in cancer cells may enhance tumor immunogenicity, excessive or non-selective inhibition risks impairing immune cell function, particularly within metabolically stressed TME [129].

Future therapeutic strategies must therefore balance tumor-directed autophagy modulation with preservation of immune competence. Cell-type-selective targeting, optimized dosing schedules, and temporally restricted intervention windows represent practical solutions to this challenge. Smart delivery platforms that preferentially release autophagy modulators within tumor cells or defined microenvironmental niches may further reduce unintended immune suppression and improve compatibility with immunotherapy.

### 7.4. Translational and Clinical Trial Challenges

Clinical trials targeting autophagy have encountered challenges related to patient selection, treatment sequencing, and toxicity management. Many early studies relied on non-specific autophagy inhibitors and lacked pharmacodynamic biomarkers to confirm effective pathway modulation, limiting interpretability and clinical impact [130]. Moreover, sustained manipulation of autophagy raises concerns regarding long-term toxicity, given its essential role in normal tissue homeostasis.

Future clinical development should emphasize rational trial design informed by tumor stage, molecular profiling, and microenvironmental characteristics. Incorporation of biomarkers to monitor autophagy flux in real time, alongside adaptive treatment strategies, may improve therapeutic precision while minimizing cumulative toxicity.

### 7.5. Future Directions: Toward Context-Aware Autophagy Therapy

The future of autophagy-targeted cancer therapy lies in embracing, rather than oversimplifying, the context-dependent nature of autophagy. Integrative strategies that combine autophagy biology, TME analysis, and advanced delivery technologies offer a path toward more effective and safer interventions.

In particular, smart and adaptive delivery systems—capable of responding to tumor-specific cues and coordinating autophagy modulation with other therapies—represent a practical route for translating autophagy knowledge into clinical benefit [131]. Advances in systems biology, artificial intelligence-driven data integration, and real-time monitoring of treatment responses may further enhance patient stratification and guide personalized intervention strategies.

Ultimately, successful translation will require interdisciplinary collaboration among basic scientists, clinicians, and bioengineers, as well as flexible therapeutic paradigms that evolve alongside tumor dynamics.

## 8. Conclusions

Autophagy is a central paradoxical process in cancer, exerting tumor-suppressive/promoting effects dependent on stage, genotype, and microenvironment. Uniform global modulation is ineffective; its clinical value lies in dynamic, context-dependent roles in tumor ecosystems. Key regulators (Beclin-1, p62/SQSTM1, mTOR, p53) integrate signals to govern tumor progression and therapy resistance; neglecting these interactions explains disappointing clinical outcomes. The TME emerges as a pivotal determinant of autophagy dependency, coordinating hypoxia-mediated metabolic adaptation, stromal support, immune modulation, and treatment-induced stress responses. Beyond facilitating tumor cell survival under adverse conditions, autophagy actively remodels tumor–immune and tumor–stroma crosstalk, highlighting the imperative of a systems-level perspective that frames autophagy as an ecosystem-wide adaptive program rather than a cancer cell-intrinsic pathway. Precision medicine offers a rational framework to dissect autophagy’s complexity in cancer by integrating tumor stage, molecular profiling, and TME features. Within this paradigm, precision nanomedicine provides robust tools for spatiotemporally controlled autophagy modulation, enabling cell-type-selective targeting while mitigating systemic toxicity and transforming autophagy intervention from a blunt strategy into a finely tunable therapeutic approach.

In conclusion, embracing autophagy’s context dependency constitutes a paradigm shift in cancer therapy. Aligning autophagy biology with TME dynamics and precision delivery technologies may enable future strategies to overcome long-standing challenges in autophagy targeting. Sustained interdisciplinary efforts are essential to translate these insights into clinically impactful, personalized cancer treatments.

## Figures and Tables

**Table 1:** Representative autophagy-modulating agents in cancer therapy.

Drug	Target	Autophagy Effect	Stage	Key Limitations
CQ	Lysosomal	Inhibition	Phase I/II	Retinal and GI toxicity; limited efficacy [61]
HCQ	Lysosomal	Inhibition	Phase I/II	Cardiotoxicity; dose limitations [55]
Rapamycin	mTORC1	Induction	clinical	Pro-survival autophagy; resistance [59]
Everolimus	mTORC1	Induction	Clinical	Metabolic side effects; resistance [62]
ULK1 inhibitors	ULK1	Inhibition	Preclinical	Limited in vivo validation [63]
VPS34 inhibitors	PI3KC3	Inhibition	Preclinical	Potential systemic toxicity [64]
Bafilomycin A1	V-ATPase	Inhibition	Preclinical	High toxicity; not clinically viable [65]

**Table 2:** Types and key characteristics of main nanocarriers.

Nanocarrier Type	Representative Examples	Key Properties	Autophagy-Related Effect	Therapeutic Outcome
Liposomes	HCQ-loaded liposomes; Rapamycin liposomes	Biocompatible; high drug loading; passive tumor accumulation (EPR)	Lysosomal inhibition or autophagy induction	Enhanced chemosensitivity; reduced systemic toxicity [70,71]
	Antibody-decorated	Surface functionalization enables cell-selective uptake	Cell-type-restricted autophagy modulation	Improved tumor specificity [72]
polymeric nanoparticles	PLGA nanoparticles carrying CQ or siRNA-BECN1	controlled release; tunable degradation	autophagy inhibition	tumor growth suppression; synergy with chemotherapy [73]
	PEGylated polymeric NPs	prolonged circulation time	sustained autophagy modulation	reduced off-target toxicity [74]
solid lipid nanoparticles (SLNs)	oligodeoxynucleotides loaded SLNs	high stability; lipophilic drug compatibility	autophagy induction	improved bioavailability and anti-tumor efficacy [75]
	SLNs for lysosomal inhibitors	enhanced intracellular delivery	autophagy flux blockade	increased therapeutic index [76]
metallic nanoparticles	gold nanoparticles (AuNPs)	easy surface modification; photothermal properties	autophagy induction or blockade (context-dependent)	photothermal-enhanced tumor cell death [77]
	gold–drug conjugates	combined physical and biochemical effects	antitumor immunotherapy by autophagy	synergistic anti-cancer activity [78]
stimuli-responsive nanoparticles	pH-responsive polymeric NPs	acidic TME-triggered release	spatially restricted autophagy modulation	enhanced efficacy in hypoxic regions [79]
	hypoxia-responsive nanocarriers	oxygen-sensitive linkers	autophagy inhibition in hypoxic niches	reduced resistance and relapse [80]
hybrid nanocarriers	liposome–polymer hybrids	structural stability and targeting flexibility	fine-tuned autophagy flux	improved combination therapy outcomes [81]

## Data Availability

No new data were created or analyzed in this study. Data sharing is not applicable to this article.
